# Neuropeptides and G-Protein Coupled Receptors (GPCRs) in the Red Palm Weevil *Rhynchophorus ferrugineus* Olivier (Coleoptera: Dryophthoridae)

**DOI:** 10.3389/fphys.2020.00159

**Published:** 2020-02-28

**Authors:** He Zhang, Juan Bai, Shuning Huang, Huihui Liu, Jintian Lin, Youming Hou

**Affiliations:** ^1^State Key Laboratory of Ecological Pest Control for Fujian and Taiwan Crops, Fujian Agriculture and Forestry University, Fujian, China; ^2^Fujian Provincial Key Laboratory of Insect Ecology, College of Plant Protection, Fujian Agriculture and Forestry University, Fujian, China; ^3^Guangzhou City Key Laboratory of Subtropical Fruit Tree Outbreak Control, Zhongkai University of Agriculture and Engineering, Guangzhou, China

**Keywords:** neuropeptides, GPCRs, immunoregulation, *Rhynchophorus ferrugineus*, expression profiling

## Abstract

The red palm weevil *Rhynchophorus ferrugineus* is a devastating, invasive pest that causes serious damages to palm trees, and its invasiveness depends on its strong ability of physiological and behavioral adaptability. Neuropeptides and their receptors regulate physiology and behavior of insects, but these protein partners have not been identified from many insects. Here, we systematically identified neuropeptide precursors and the corresponding receptors in the red palm weevil, and analyzed their tissue expression patterns under control conditions and after pathogen infection. A total of 43 putative neuropeptide precursors were identified, including an extra myosuppressin peptide was identified with amino acid substitutions at two conserved sites. Forty-four putative neuropeptide receptors belonging to three classes were also identified, in which neuropeptide F receptors and insulin receptors were expanded compared to those in other insects. Based on qRT-PCR analyses, genes coding for several neuropeptide precursors and receptors were highly expressed in tissues other than the nervous system, suggesting that these neuropeptides and receptors play other roles in addition to neuro-reception. Some of the neuropeptides and receptors, like the tachykinin-related peptide and receptor, were significantly induced by pathogen infection, especially sensitive to *Bacillus thuringiensis* and *Metarhizium anisopliae*. Systemic identification and initial characterization of neuropeptides and their receptors in the red palm weevil provide a framework for further studies to reveal the functions of these ligand- and receptor-couples in regulating physiology, behavior, and immunity in this important insect pest species.

## Introduction

Neuropeptides are a class of signal molecules secreted by neuroendocrine cells for regulating the transmission of intercellular signals. Neuropeptides regulate behavioral activities of insects via their interactions with the corresponding receptors and subsequent signal transduction, and those behavioral activities can be further categorized into behaviors (involving feeding, reproduction, learning and memory, stress and addiction, circadian rhythms, sleep, wakefulness, social behavior) and physiological processes (including growth and development, digestion, energy homeostasis, water and ion balance, and metabolism) (Caers et al., 2012; Schoofs et al., 2017; Yeoh et al., 2017; Nässel and Zandawala, 2019). Neuropeptides are usually produced from the cleavage of larger precursors and are usually modified post-transcriptionally to form isopeptides, which are then transported to target cells to activate corresponding receptors (Veenstra, 2000; Pauls et al., 2014; Yeoh et al., 2017). A large number of neuropeptides and their receptors have been extensively characterized and functionally validated in various insects, such as *Drosophila melanogaster* (Nässel and Winther, 2010), *Tribolium castaneum* (Li et al., 2007; Hauser et al., 2008), *Locusta migratoria* (Veenstra, 2014; Hou et al., 2015), and *Bombyx mori* (Roller et al., 2008). These results suggest significant neuropeptide and receptor variation between different orders, even between different species from the same order (Veenstra, 2019). Insect neuropeptides and their GPCRs are promising targets for a novel generation of pesticides. Thus, identification and functional characterization of neuropeptides and receptors from insect pests may provide useful information for pest management and for enhancing our basic understanding of neuropeptide-related signal transduction.

The red palm weevil *Rhynchophorus ferrugineus* (Coleoptera: Curculionidae) is a devastating pest, which has been spread to various regions with palm trees in southern China, causing serious damage to the palm industry and landscape (Hou et al., 2011; Wang et al., 2015, 2017; Muhammad et al., 2017; Ali et al., 2018). Red palm weevil larvae have a long life span, strong ability to drill collar, and strong adaptability to different environments (Shi et al., 2014; Peng et al., 2016; Dawadi et al., 2018; Habineza et al., 2019; Xiao et al., 2019). Due to damage to vascular tissues and consumption of large amounts of crown tissues, red palm weevil larvae can cause the death of palm trees (Butera et al., 2012; Muhammad et al., 2017). When symptom appears, it is usually too late to save the trees (Peng and Hou, 2017). Therefore, it is urgent to develop novel technologies for controlling this destructive pest at an early stage to reduce damage to palm trees (Pu and Hou, 2016; Pu et al., 2017). Neuropeptides and their receptors, the important behavioral and physiological regulators of insects, might be potential targets for developing novel methods for pest control (Audsley and Down, 2015). Very limited information is available on neuropeptides and their functions on the red palm weevil. Base on genomics, transcriptomics, and peptidomics, numerous neuropeptides and receptors have been identified from Coleopterans (Weaver and Audsley, 2008; Cunningham et al., 2017; Pandit et al., 2018; Veenstra, 2019). The types and numbers of neuropeptides and receptors in Coleopterans are quite different from those from other insect species. Significant variation has also been observed even among species within the order Coleoptera (Veenstra, 2019). For those identified genes, tissue and developmental expression profiles have been used in many insect species to mine for functional information (Hou et al., 2015; Xu et al., 2016; Wang et al., 2018). Identification and expression analyses of neuropeptides and corresponding receptors from the red palm weevil should provide useful information for comparative studies and exploration of practical application.

In addition to regulating various physiology and behavior of insects, neuropeptides may participate in immune responses of insects (Urbanski and Rosinski, 2018). One of the reasons for the red palm weevil succeeds in spreading globally is its strong immunity to pathogen’s attack. It would be interesting to explore any role of neuropeptides in immunity of the red palm weevil. In this study, we have systematically identified neuropeptides and their receptors in the red palm weevil following a transcriptomic approach. We then analyzed sequence variation and phylogenetic relationship of the identified neuropeptides and receptors together with those identified from other insect species previously. Tissue expression profiles of neuropeptide precursors and corresponding receptors were examined via quantitative real time-PCR (qRT-PCR). Potential impact of pathogenic microbes on the expression of the newly identified neuropeptide precursors and corresponding receptors was also examined.

## Materials and Methods

### Insect Rearing

The colony of the red palm weevil used in this study was originated from adults trapped from the campus of Fujian Agriculture and Forestry University in September 2017. The colony has been maintained in incubators since then. Adult males and females were fed with sugarcane stems in pairs at 27 ± 1°C, 75% relative humidity (RH), and a light: dark cycle of 12:12. Eggs were regularly collected on wet filter papers. The collected eggs were used to inoculate cuts of sugarcane stems right before larval hatch. Larvae were reared individually with regular diet changed until pupation and emergence.

### Identification of the Neuropeptides and Their Putative G Protein-Coupled Receptors

The transcriptomic (RNA-seq) data derived from larvae and pupae of the red palm weevil were used for identification of the neuropeptides and receptors. Larvae data was downloaded from the published database (NCBI Sequence Read Archive: SRX096969), and pupae data was obtained from our laboratory (unpublished data). After assembly, unigenes encoding neuropeptides and receptors were identified by BLAST searches against a local database with amino acid sequences of the neuropeptide precursors and receptors of *D. melanogaster*, *T. castaneum*, *Hylobius abietis*, and other insects as queries. The cut-off Expectation Value (E) threshold was 1.0 for putative neuropeptides, and 0.001 for receptors. Candidate genes identified from searches were further verified by additional BLAST searches against the NCBI non-redundant protein database (BLASTx) to remove false positives and repeat sequences.

### Structural, Domain, and Sequence Analyses

Open reading frames (ORFs) of candidate genes were predicted using the NCBI ORF finder^1^. Secretion signal peptides were identified using SignalP 4.0^2^ (Petersen et al., 2011). Sequence logos for neuropeptide motifs were analyzed using Weblogo^3^ (Crooks et al., 2004). Sequences for multiple sequence alignments were downloaded from DINeR^4^ (Yeoh et al., 2017). Multiple alignments of amino acid sequences were performed with MAFFT (Katoh and Standley, 2013), and visualized with Jalview 2.10.3 (Waterhouse et al., 2009).

### Phylogenetic Analysis

Amino acid sequences used for phylogenetic tree construction were aligned with the MAFFT (Katoh and Standley, 2013). Phylogenetic trees were constructed with FastTree (version 2.1.7) using the maximum-likelihood method with 1000 bootstrap replicates (Price et al., 2010). Phylogenetic trees were edited and visualized with FigTree 1.4.4^5^.

### Tissue Expression Analysis of Neuropeptide Precursors and Receptors

To examine tissue expression profiles of neuropeptide precursors and their putative receptors, total RNA was extracted from various tissues, including the hemocytes (HC), fat body (FB), gut (including the foregut, midgut, hindgut, Malpighian tubes) and CNS (including the brain and ventral nerve cord) from eighth instar larvae using TRIzol reagent (Invitrogen, United States) following the manufacturer’s instructions. The hemocytes were collected according to the method for studying *Chilo suppressalis* (Xu et al., 2016). cDNA was synthesized using a PrimeScript^TM^ RT reagent Kit with gDNA Eraser (Perfect Real Time) (Takara, China). Primers specific to individual genes for qRT-PCR analyses were designed using the Primer 3 program^6^ and are listed in Supplementary Table S5. The RT-qPCR experiments were performed according to the Minimum Information Required for Publication of Quantitative Real-Time PCR Experiments (MIQE) Guidelines (Johnson et al., 2014). qRT-PCR were performed on a Light Cycler 480 System (Roche Applied Science) using a 10 μl reaction containing 5 μl 2 × SYBR Green PCR Master Mix (Roche, Germany), 0.5 μl of each primer (10 μM), 1 μl of cDNA template, and 3 μl of sterile H_2_O. PCR reactions were proceeded at 95°C for 15 min, followed by 40 cycles of 95°C for 10 s and 60°C for 32 s. Dissociation curves for PCR products were generated by heating to 95°C for 15 s, followed by cooling to 60°C for 1 min; heating to 95°C for 30 s, followed by cooling to 60°C for 15 s. Each sample had four biological replicates and each replicate had three technical duplicates. Relative transcript abundance was determined using the 2^–Δ*CT*^ method (Schmittgen and Livak, 2008), with the red palm weevil *gapdh* and *tubulin* genes as an internal reference. Comparative analyses of each target gene among different tissues were determined using a one-way nested analysis of variance (ANOVA) followed by a least significant difference test (LSD) for mean comparison, and data analyses were done in Prism7.0 (GraphPad Software, San Diego, CA, United States). Heatmaps of gene expression for different neuropeptides and their receptors genes among different tissues were generated by R version 3.4.1.

### Impact of Pathogens on Expression of Neuropeptide and Receptor Genes

Three pathogens were selected to examine their impact on the expression of genes coding for neuropeptides and receptors. These pathogens were *Serratia marcescens* (Gram−), *Bacillus thuringiensis* (Gram+), and *Metarhizium anisopliae* (Fungus). PBS was used as control. The bacteria *S. marcescens* and *B. thuringiensis* were cultured overnight on Nutrient Broth (NB) at 28°C in a shaker at 200 rpm. Bacterial cells were harvested by centrifuging and washed three times with sterilized PBS by re-suspending in PBS. *M. anisopliae* spores were scraped from PDA medium, dissolved in PBS, and filtered through sterile gauze to obtain a fungal spore suspension. Cell density of the three pathogens were estimated using a hemocytometer and adjusted to required density with PBS. Early third-instar larvae of the red palm weevil with an average weight of 150 mg were chosen for infection. Larvae were surface-sterilized with 70% ethanol before pathogen injection. Larvae were randomly selected and individually injected with 5 μl PBS (control), or 5 μl PBS containing either 1 × 10^4^ *S. marcescens*, 1 × 10^2^ *B. thuringiensis* cells, or 1 × 10^2^ *M. anisopliae* spores. Solution was injected into the hemocoel of each larva via the last left proleg. After injection, insects were collected for RNA extraction at different time points, including 0, 3, 6, 12, 48 h. Four larvae at each time point were combined for RNA extraction in each sample. Four biological replicates were included in each time point. RNA extraction, cDNA synthesis, qRT-PCR and quantification of transcript abundance were carried out as described in earlier sections. Comparative analyses of each target gene among different time points for the same treatment and the same time point among different treatments were conducted using a one-way nested analysis of variance (ANOVA) followed by a LSD for mean comparison. All data analyses were done in Prism7.0 (GraphPad Software, San Diego, CA, United States).

## Results

### Overview of Transcriptomes

We generated two transcriptomes, one from larvae and the other from pupae. The raw reads of larval was downloaded from NCBI Sequence Read Archive (SRX096969) for reassembly and annotation. A total of 74.9 million raw reads were obtained from the pupal transcriptome. After removing low quality reads, adaptor sequences, and reads shorter than 20 bp, the remaining high-quality reads were 73.0 million for pupal transcriptome. High quality reads were then assembled into unigenes separately. A total of 16,875 unigenes were obtained for the larval transcriptome, with the average length 1138 bp and N50 1427. A total of 37,210 uigenes were obtained for the pupal transcriptome, with the average length 2025 bp and N50 3320. Unigenes from both transcriptomes were used to identify neuropeptide and receptor genes.

### Identification of Various Neuropeptides

A total of 43 transcripts encoding putative neuropeptide precursors and putative neuropeptides from precursors were identified from the red palm weevil transcriptomes (Table 1 and Supplementary Tables S1, S3). Among them, 29 transcripts encode full length proteins, and most of the predicted proteins contain a signal peptide. The identified neuropeptide precursors share sequence similarity with homologs from *T. castaneum*, *Dendroctonus ponderosae*, and *Hylobius abietis*.

**TABLE 1:** Neuropeptide precursors identified from the red palm weevil.

					**Homology search with known protein**		
**Neuropeptide**	**Accession no.**	**Abbreviation**	**ORF (aa)**	**SP (aa)**	**Species**	**Protein ID**	**E-Value**
Allatostatin B	MK751535	Ast B	199	37	*Asbolus verrucosus*	RZC34805.1	3e−44
Allatostatin C	MK751536	Ast C	114^a^	26	*Tribolium castaneum*	EFA09152.2	4e−24
Allatostatin CC	MK751537	AstCC	71^a^	∼	*Tribolium castaneum*	XP_001810067.1	7e−15
Allatotropin	MK751538	AT	84^a^	∼	*Hylobius abietis*	SRP133355	2e−29
Bursicon	MK751539	Burs	80^a^	∼	*Dendroctonus ponderosae*	XP_019755391.1	8e−49
Calcitonin	MK751540	Cal	283	20	*Hylobius abietis*	SRP133355	3e−66
Capability/CAP2b	MK751541	CAPA	195	23	*Hylobius abietis*	SRP133355	2e−41
CCHamide−1	MK751542	CCHa-1	116^a^	33	*Tribolium castaneum*	XP_008201341.1	4e−10
CCHamide−2	MK751543	CCHa-2	118	28	*Tribolium castaneum*	XP_008190391.1	5e−11
CNMamide	MK751544	CNMa	130	21	*Hylobius abietis*	SRP133355	3e−07
Diuretic Hormone 31	MK751545	DH31	121	32	*Anoplophora glabripennis*	XP_018565090.1	8e−34
Diuretic hormone 44	MK751546	DH44	197	∼	*Dendroctonus ponderosae*	XP_019767380.1	7e−69
Ecdysis triggering hormone	MK751547	ETH	130	17	*Leptinotarsa decemlineata*	QBH70331.1	8e−19
Eclosion hormone	MK751548	EH	49^a^	∼	*Leptinotarsa decemlineata*	XP_023028729.1	8e−20
Elevenin	MK751549	Ele	100^a^	∼	*Dendroctonus ponderosae*	XP_019770585.1	2e−04
FMRFamide	MK751550	FMRFa	167^a^	18	*Dendroctonus ponderosae*	XP_019753890.1	8e−19
	MK751551						
Glycoprotein hormone alpha 2	MK751552	GPA2	122	16	*Asbolus verrucosus*	RZC35091.1	6e−54
Glycoprotein hormone beta 5	MK751553	GPA5	154^a^	18	*Tribolium castaneum*	NP_001280517.1	1e−73
locust insulin-related peptide	MK751554	LIRP	115	29	*Anoplophora glabripennis*	XP_018573725.1	4e−08
Ion transport peptide	MK751555	ITPa	96	31	*Anoplophora glabripennis*	XP_018574385.1	1e−30
Ion transport peptide	MK751556	ITPb	135	31	*Asbolus verrucosus*	RZC33292.1	1e−57
ITG-like peptide	MK751557	ITG	213	19	*Dendroctonus ponderosae*	XP_019771238.1	2e−110
Myosuppressin	MK751558	MS-1	95	24	*Anoplophora glabripennis*	XP_018573133.1	1e−25
Myosuppressin	MK751559	MS-2	91	24	*Anoplophora glabripennis*	XP_018573133.1	3e−14
Natalisin	MK751560	NTL	155	34	*Tribolium castaneum*	XP_015833286.1	1e−21
Neuroparsin	MK751561	NP	102	21	*Anoplophora glabripennis*	XP_018568084.1	2e−31
Neuropeptide F (long transcript)	MK751562	NPFa	129	25	*Dendroctonus ponderosae*	XP_019762446.1	1e−53
Neuropeptide F (short transcript)	Unigene0020442	NPFb	92	25			
Neuropeptide-like precursor 1a	MK751563	NPLP1a	308	27	*Dendroctonus ponderosae*	XP_019771539.1	6e−106
Neuropeptide-like precursor 1b	MK751564	NPLP1b	282	27	*Dendroctonus ponderosae*	XP_019771539.1	5e−84
NVP-like	MK751565	NVP	327	17	*Tribolium castaneum*	XP_008196925.1	3e−78
Orcokinin B	MK751566	OK-B	357	19	*Hylobius abietis*	SRP133355	2e−21
Pigment dispersing factor	Unigene0014866	PDF	105	30	*Sitophilus oryzae*	XP_030754781.1	5e−43
Proctolin	MK751567	Proc	90	26	*Dendroctonus ponderosae*	XP_019763695.1	1e−09
Prothoracicotropic hormone	MK751568	PTTH	82^a^	∼	*Rhynchophorus ferrugineus*	ATU47262.1	3e−43
Pyrokinin 1	MK751569	PK1	147^a^	26	*Dendroctonus ponderosae*	XP_019770936.1	5e−17
Pyrokinin 2	MK751570	PK2	105^a^	∼	*Dendroctonus ponderosae*	XP_019770936.1	8e−12
RYamide	MK751571	RYa	117	25	*Asbolus verrucosus]*	RZC32873.1	2e−23
Short neuropeptide F	MK751572	sNPF	100	25	*Dendroctonus ponderosae*	XP_019767920.1	1e−37
Sulfakinin	MK751573	SK	112	32	*Tribolium castaneum*	EFA04708.1	7e−16
Tachykinin-related peptide	MK751574	TRP	264	25	*Dendroctonus ponderosae*	XP_019770548.1	1e−91
Trissin	MK751575	Tris	87^a^	∼	*Dendroctonus ponderosae*	XP_019766470.1	8e−39
Vasopressin	MK751576	VPL	142	26	*Tribolium castaneum*	NP_001078831.1	1e−35

*ORF, open reading frame; SP, signal peptide; ^*a*^not full length; ∼no signal peptide.*

**FIGURE 1:**
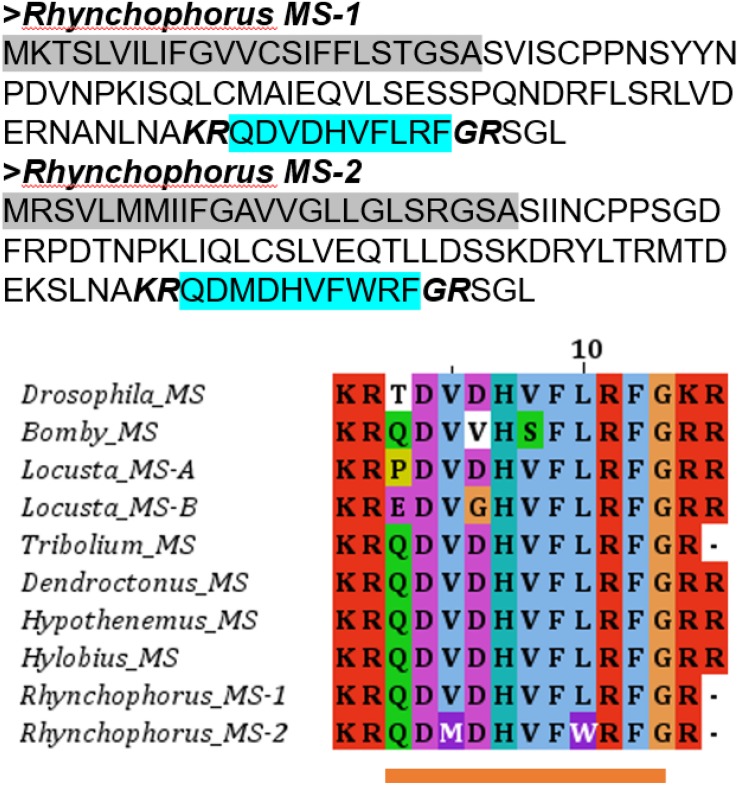
Myosuppressin precursors of the red palm weevil and multiple sequence alignment of myosuppressin peptide of the red palm weevil with other insect species. Predicted signal peptides (highlighted in gray), cleavage signals (italics, bold) and supposed bioactive mature peptides are indicated. The sequence underlined in orange is the predicted mature peptide.

Two transcripts encoding two different myosuppressin precursors, MS-1 and MS-2, were identified, with each encoding a different myosuppresin peptide. MS-1 and MS-2 differ significantly in amino acid sequence in both signal peptide- and mature peptide-coding regions (Figure 1). A sequence alignment of MS-1 and MS-2 together with the corresponding sequences from other insects revealed that MS-1 is identical to the myosuppresin from the beetles *T. castaneum*, *D. ponderosae*, and *H. abietis*. However, amino acid substitutions occurred at the 3rd and 8th positions of MS-2, with Val at the third position and Leu at the eighth position replaced with Met and Trp, respectively, in MS-2 (Figure 1).

Two neuropeptide F (NPF) transcripts of different lengths produced by alternative splicing were identified in red palm weevil (Supplementary Figure S1), consistent with NPF precursors found in other insects (Nässel and Wegener, 2011; Veenstra, 2014). The long splice variant of the *npf* gene has an optional exon compared to the short splice variant (Supplementary Figure S1). However, it seems that only mature neuropeptides predicted from the short splice variant of *npf* gene have been identified in neuropeptidomic of these insects (Nässel and Wegener, 2011; Pandit et al., 2018), whether long splice variant of *npf* gene encodes different mature neuropeptide remains unclear.

The Pigment dispersing factor (PDF) has undergone significant sequence changes in Coleoptera, especially in its C-terminal half (Veenstra, 2019). According to this, a transcript encoding the neuropeptide PDF was identified from the red palm weevil transcriptomes. An sequence alignment revealed that the predicted PDF from the red palm weevil showed high sequence similarity to those from Coleopterans (Veenstra, 2019), but lost the Arg- Lys cleavage sites at the C-terminal (Supplementary Figure S2).

Orcokinins were initially isolated from *Orconectes limosus* and have also been generally identified from insects (Stangier et al., 1992; Veenstra, 2014). In *T. castaneum*, two isoforms, named orcokinin-A (OK-A) and orcokinin-B (OK-B), have been identified, and are encoded by the same gene through alternative splicing (Jiang et al., 2015). Here we identified one orcokinin, which is similar to the B form. The orcokinin B precursor can be processed into several similar isopeptides, and the number of peptides varies from species to species. The number of iso-orcokinin-B is predicted 22 for the red palm beetle, more than 15 in *H. abietis* (Pandit et al., 2018), 10 in *T. castaneum* (Jiang et al., 2015), and only one in *D. melanogaster* (Veenstra and Ida, 2014). The consensus for Orcokinin B isopeptides is X(I, L, V)DXXGGG in N-terminal based on sequence alignments (Supplementary Figure S3).

Calcitonin-like diuretic hormone plays a role in regulating salt and water transport of insects and is considered to be the insect calcitonin ortholog (Zandawala, 2012; Veenstra, 2014). Calcitonin-like diuretic hormone is ubiquitous in insects, but calcitonin is only reported in several insects (Yeoh et al., 2017). Calcitonin can be separated into two distinct classes, calcitonin-A and calcitonin-B. In the red palm weevil, a transcript encoding calcitonin-B was identified. In both *T. castaneum* and *H. abietis*, two genes coding for calcitonin-B produce four and six isopeptides, respectively (Veenstra, 2014; Pandit et al., 2018). The red palm weevil transcript encodes seven mature isopeptides (Supplementary Table S3).

The number of insulin-like peptide (ILPs), also known as insulin-related peptide (IRP), varies widely in different insect species. For example, there are 50 in the silkworm (Aslam et al., 2011; Mizoguchi and Okamoto, 2013), but only one in the migratory locust (Veenstra, 2014). In *T. castaneum*, there are four insulin-like peptide-encoding genes assigned to three evolutionary groups based on their conserved motif (Li et al., 2007). Here in the red palm weevil, only one insulin gene was found, and the gene encodes a peptide similar to the *T. castaneum* ILP-B, with a CCxxxC motif. In Coleoptera, the number of insulin genes range from 2 to 10 and varies significantly in sequence (Veenstra, 2019). Our transcriptomes may not cover all of the insulin genes in the red palm weevil.

A pyrokinin and Capa precursor in hexapods can result in three types of neuropeptides: periviscerokinins (PVKs), pyrokinins (PKs), and tryptoPKs, and each one activates a specific receptor (Terhzaz et al., 2012; Pandit et al., 2018). The number and combination of these three peptides differ in different species based on different ways of gene duplication and subsequent diversification (Derst et al., 2016). In the red palm weevil, transcripts encoding one complete capa and two incomplete pyrokinin precursors were identified, similar to that reported in several tenebrionid beetles (Neupert et al., 2018; Pandit et al., 2018). The CAPA precursor from the red palm weevil contains at least two potential PVKs and a single tryptoPK. Among the two PK genes, one coding for a PK precursor that can produce three pyrokinins and one tryptoPK, and the other coding for a precursor that can produce two pyrokinins and one tryptoPK (Supplementary Table S3).

In addition, we identified two CCHamides, two ion transport peptides and two neuropeptide-like precursors from the red palm weevil transcriptomes. The two ion transport peptides and the two neuropeptide-like precursors are produced by alternative splicing and identical in sequence, but varied in sequence length. However, the two CCHamide precursors encode different types of peptides: CCHa-1 and CCHa-2. Multiple sequence alignments indicate that most insect mature CCHa-1 peptides have the consensus SCLSYGHSCWGAH, and CCHa-2 have the consensus GCSXFGHSCFG(G,A)H. However, the conserved Ser at position 8 of CCHa-1 is mutated to Ala in all Cucujiformia beetles, while the conserved Gly-His-amide at C-terminal of CCHa-2 is mutated to Gly-Met-amide in most Coleoptera beetles, the red palm weevil is even more strangely mutated to Ala-Leu-amide (Supplementary Figures S4, S5).

No transcripts encoding adipokinetic hormone, AKH/Corazonin-related peptide, Agatoxin-like, crustacean cardio-active peptide, Hansolin, Relaxin, RFLamide, and SIFamide were found in the transcriptomes of the red palm weevil (Supplementary Table S4).

### Identification of G Protein-Coupled Receptors (GPCRs) for Neuropeptides

A total of 44 putative neuropeptide GPCRs were identified from the red palm weevil transcriptomes (Table 2 and Supplementary Table S2). These GPCRs were divided into three classes, 30 belonged to the Class A (Rhodopsin-like receptor family), five belonged to the Class B (Secretin-like receptor family), and the remaining nine belonged to LGRs (Leucine-rich repeat-containing GPCRs).

**TABLE 2:** Putative G protein-coupled receptors for neuropeptides identified from the red palm weevil.

				**Homology search with known protein**		
		**GPCR**	**ORF**			
**Neuropeptide receptor**	**Accession no.**	**Class/Type**	**(aa)**	**Species**	**Protein ID**	**E-Value**
Adipokinetic hormone	MK751489	Class A	377	*Dendroctonus ponderosae*	XP_019759039.1	0.0
Allatostatin C	MK751490	Class A	426	*Dendroctonus ponderosae*	XP_019756512.1	0.0
Capability	MK751491	Class A	266^a^	*Dendroctonus ponderosae*	XP_019756922.1	3e−132
CCHamide	MK751492	Class A	340^a^	*Dendroctonus ponderosae*	XP_019758999.1	0.0
Crustacean CardioActive Peptide	MK751493	Class A	152^a^	*Tribolium castaneum*	XP_015837746.1	1e−50
Ecdysis-triggering hormone	MK751494	Class A	465	*Dendroctonus ponderosae*	XP_019769415.1	0.0
FMRFamide receptor	MK751495	Class A	438	*Dendroctonus ponderosae*	XP_019768282.1	0.0
Long neuropeptide F	MK751496	Class A	449	*Leptinotarsa decemlineata*	XP_023018238.1	7e−174
Neuropeptide F 1	MK751497	Class A	414	*Dendroctonus ponderosae*	XP_019756679.1	0.0
Neuropeptide F 2	MK751498	Class A	429	*Dendroctonus ponderosae*	XP_019756688.1	0.0
Orphan1	MK751499	Class A	553	*Dendroctonus ponderosae*	XP_019754524.1	0.0
Orphan2	MK751500	Class A	447	*Sitophilus oryzae*	ALM55746.1	0.0
Orphan3	MK751501	Class A	342	*Anoplophora glabripennis*	XP_018566563.1	1e−174
Orphan4	MK751502	Class A	339	*Dendroctonus ponderosae*	XP_019754869.1	0.0
Proctolin	MK751503	Class A	552	*Dendroctonus ponderosae*	XP_019770777.1	0.0
Pyrokinin 1	MK751504	Class A	490	*Dendroctonus ponderosae*	XP_019763370.1	0.0
Pyrokinin 2	MK751505	Class A	526	*Dendroctonus ponderosae*	XP_019762501.1	0.0
RFLa peptide	MK751506	Class A	392	*Dendroctonus ponderosae*	XP_019764941.1	0.0
Sex peptide 1	MK751507	Class A	437	*Dendroctonus ponderosae*	XP_019758277.1	1e−166
Sex peptide 2	MK751508	Class A	380	*Dendroctonus ponderosae*	XP_019759112.1	0.0
Sex peptide 3	MK751509	Class A	317^a^	*Dendroctonus ponderosae*	XP_019769212.1	4e−101
	MK751510					
Sex peptide 4	MK751511	Class A	426^a^	*Dendroctonus ponderosae*	XP_019770807.1	3e−170
	MK751512					
Short neuropeptide F	MK751513	Class A	426	*Dendroctonus ponderosae*	XP_019761868.1	0.0
SIFamide	MK751514	Class A	446	*Dendroctonus ponderosae*	XP_019765870.1	2e−180
Sulfakinin	MK751515	Class A	424	*Dendroctonus ponderosae*	XP_019756917.1	0.0
Tachykinin-related peptide	MK751516	Class A	422	*Dendroctonus ponderosae*	XP_019768744.1	0.0
Trissin1	MK751517	Class A	410	*Dendroctonus ponderosae*	XP_019773394.1	0.0
Trissin2	MK751518	Class A	241^a^	*Dendroctonus ponderosae*	XP_019759003.1	5e−105
Inotocin (vasopressin-like)	MK751519	Class A	375	*Tribolium castaneum*	XP_015837046.1	0.0
Orphan5	MK751520	Class A	492	*Dendroctonus ponderosae*	XP_019761979.1	0.0
Calcitonin	MK751521	Class B	541	*Dendroctonus ponderosae*	XP_019756394.1	0.0
Diuretic hormone 31	MK751522	Class B	286^a^	*Dendroctonus ponderosae*	XP_019763869.1	3e−124
Diuretic hormone 44	MK751523	Class B	415	*Dendroctonus ponderosae*	XP_019758310.1	3e−90
Pigment-dispersing factor	MK751524	Class B	469	*Dendroctonus ponderosae*	XP_019773389.1	2e−162
Orphan6	MK751525	Class B	453	*Dendroctonus ponderosae*	XP_019757091.1	7e−144
Leucine-rich repeat-containing GPCR – FSH	MK751526	Type A	769	*Dendroctonus ponderosae*	XP_019769811.1	0.0
Leucine-rich repeat-containing GPCR – LGR4-like	MK751527	Type B	598	*Dendroctonus ponderosae*	XP_019753406.1	5e−153
Leucine-rich repeat-containing GPCR – Burs-like	MK751528	Type B	1169^a^	*Tribolium castaneum*	EFA02891.2	0.0
Leucine-rich repeat-containing GPCR – LGR5-like1	MK751529	Type B	672	*Dendroctonus ponderosae*	XP_019754543.1	0.0
Leucine-rich repeat-containing GPCR – LGR5-like2	MK751530	Type B	495	*Dendroctonus ponderosae*	XP_019760595.1	0.0
Leucine-rich repeat-containing GPCR – insulin-like1	MK751531	Type C	1391	*Dendroctonus ponderosae*	XP_019765260.1	0.0
Leucine-rich repeat-containing GPCR – insulin-like2	MK751532	Type C	1226	*Dendroctonus ponderosae*	XP_019755840.1	0.0
Leucine-rich repeat-containing GPCR – insulin-like3	MK751533	Type C	1319	*Dendroctonus ponderosae*	XP_019761347.1	0.0
Leucine-rich repeat-containing GPCR – relaxin 2-like	MK751534	Type C	424^a^	*Dendroctonus ponderosae*	XP_019760354.1	1e−160

**FIGURE 2:**
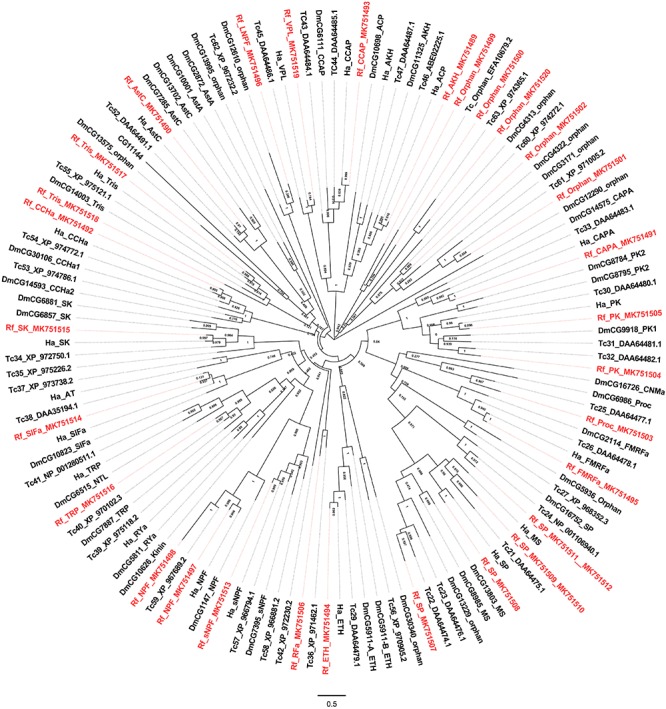
Phylogenetic tree analysis of the Class A neuropeptide GPCRs from *Rhynchophorus ferrugineus* (Rf), *H. abietis* (Ha), *T. castaneum* (Tr), *Drosophilla melanogaster* (Dm).

Class A contains the most receptors, and their potential neuropeptide ligands include Adipokinetic hormone, Allatostatin C, CCHamide 1, Ecdysis-triggering hormone, etc. A phylogenetic analysis showed that class A receptors from the red palm weevil are clustered with counterparts from other insect species including *T. castaneum*, *D. melanogaster*, and *H. abietis* (Figure 2) (Hauser et al., 2008; Pandit et al., 2018). GPCRs not found in the red palm weevil (but their corresponding neuropeptides were identified) included those receptors for allatotropin, CCHamide2, myosuppressin, and RYamide. GPCRs for adipokinetic hormone, the crustacean cardioactive peptide, RFa peptide, sex peptide, and SIFamide were identified from the red palm weevil. However, their corresponding neuropeptides were not found yet. The GPCRs for sex peptide, neuropeptide F, and trissin appeared to have expanded in the red palm weevil. Specifically, there were four sex peptide receptors, three receptors for neuropeptide F (including long neuropeptide F, neuropeptide F1, neuropeptide F2), and two trissin receptors identified in the red palm weevil, compared with only one sex peptide receptor, one neuropeptide F receptors, and one trissin receptor has been reported in *H. abietis*. In addition, five orphan GPCRs were identified from the red palm weevil, but their neuropeptide ligands remained unknown.

**FIGURE 3:**
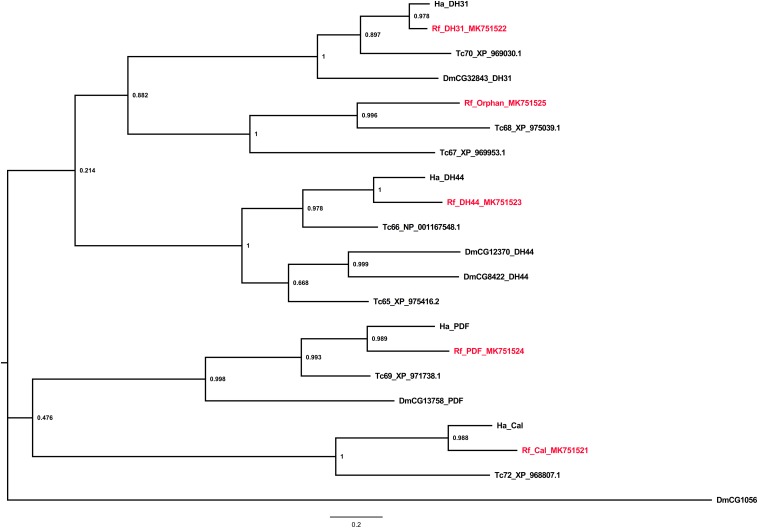
Phylogenetic tree analysis of the Class B neuropeptide GPCRs from *R. ferrugineus*, *H. abietis*, *T. castaneum*, *D. melanogaster*.

Class B receptors for calcitonin, diuretic hormones 31 and 44, and pigment-dispersing factor were identified along with an orphan GPCR from the red palm weevil. All the identified receptors were found to share evolutionary relationship (Figure 3).

**FIGURE 4:**
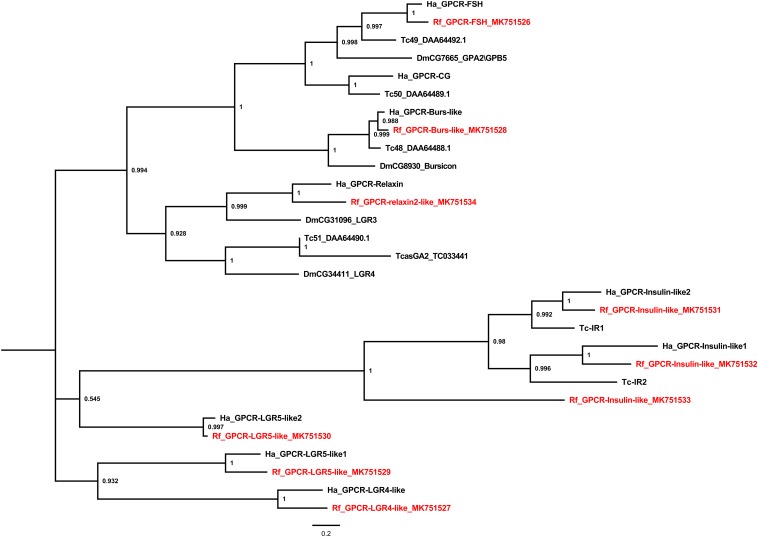
Phylogenetic tree analysis of the Leucine-rich Repeat-containing GPCRs (LGR) from *R. ferrugineus*, *H. abietis*, *T. castaneum*, *D. melanogaster*.

Several leucine-rich repeats-containing GPCRs (LGRs) were also identified (Figure 4). The LGRs were divided into three types according to the numbers of leucine-rich repeat motifs, types A, B, and C. Type-A LGRs include follicle stimulating hormone receptor (FSH) and the choriogonadotropin receptor. However, no choriogonadotropin receptor was found in the red palm weevil. Type-B LGRs include Bursicon-like as well as LGR4 and 5-like receptors, all of which were identified in the red palm weevil. Type-C LGRs identified from the red palm weevil include one relaxin 2-like and three insulin-like receptors, which is unusual since Coleopterans, Hymenopterans, and Hemipteran were thought to have only two insulin receptors (Sang et al., 2016), as is the case in *T. castaneum* and *H. abietis* (Figure 4).

### Expression Profiles of the Neuropeptides and Neuropeptide Receptors

**FIGURE 5:**
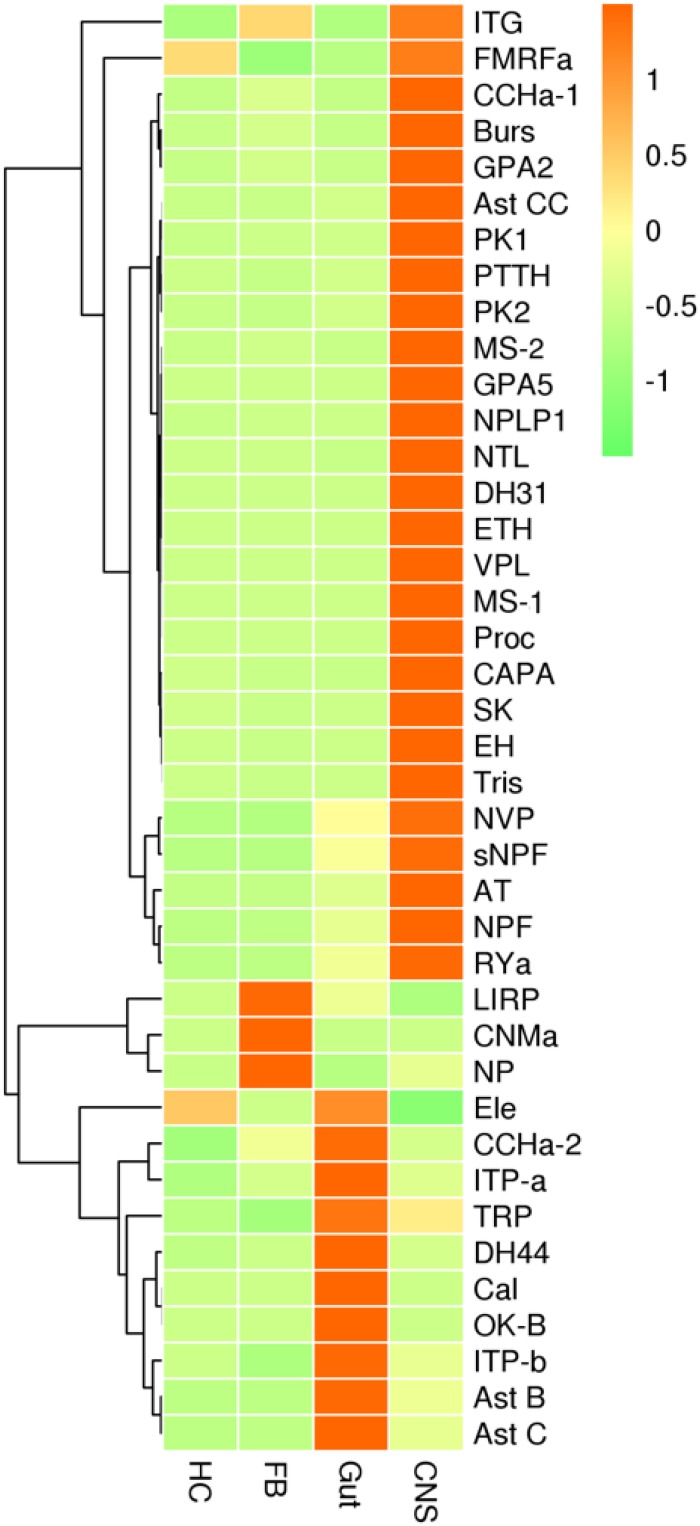
qRT-PCR results showing the relative expression levels of the neuropeptide precursors in various tissues of the red palm weevil. HC, hemocytes; FB, fat bodies; Gut, including foregut, midgut, hindgut, and Malpighian tubes; CNS, central nervous system. The expression levels were estimated using the 2^– Δ^ ^CT^ method. Red indicates overexpression, while green represents low expression.

Gene expression profiles were analyzed in four types of tissues, including the central nervous system (CNS) (brain and ventral nerve cord), the gut (foregut, midgut, hindgut, and Malpighian tubes), hemocytes, and fat bodies. Most neuropeptide precursors were expressed at the highest levels in CNS. The genes coding for allatostatin B, allatostatin C, Calcitonin, diuretic hormone 44, Ion transport peptide a and b, orcokinin B were mainly expressed in gut plus Malpighian tubules. The genes coding for CNMamide, insulin-related peptide, neuroparsin were mainly expressed in fat bodies (Figure 5 and Supplementary Figure S6).

**FIGURE 6:**
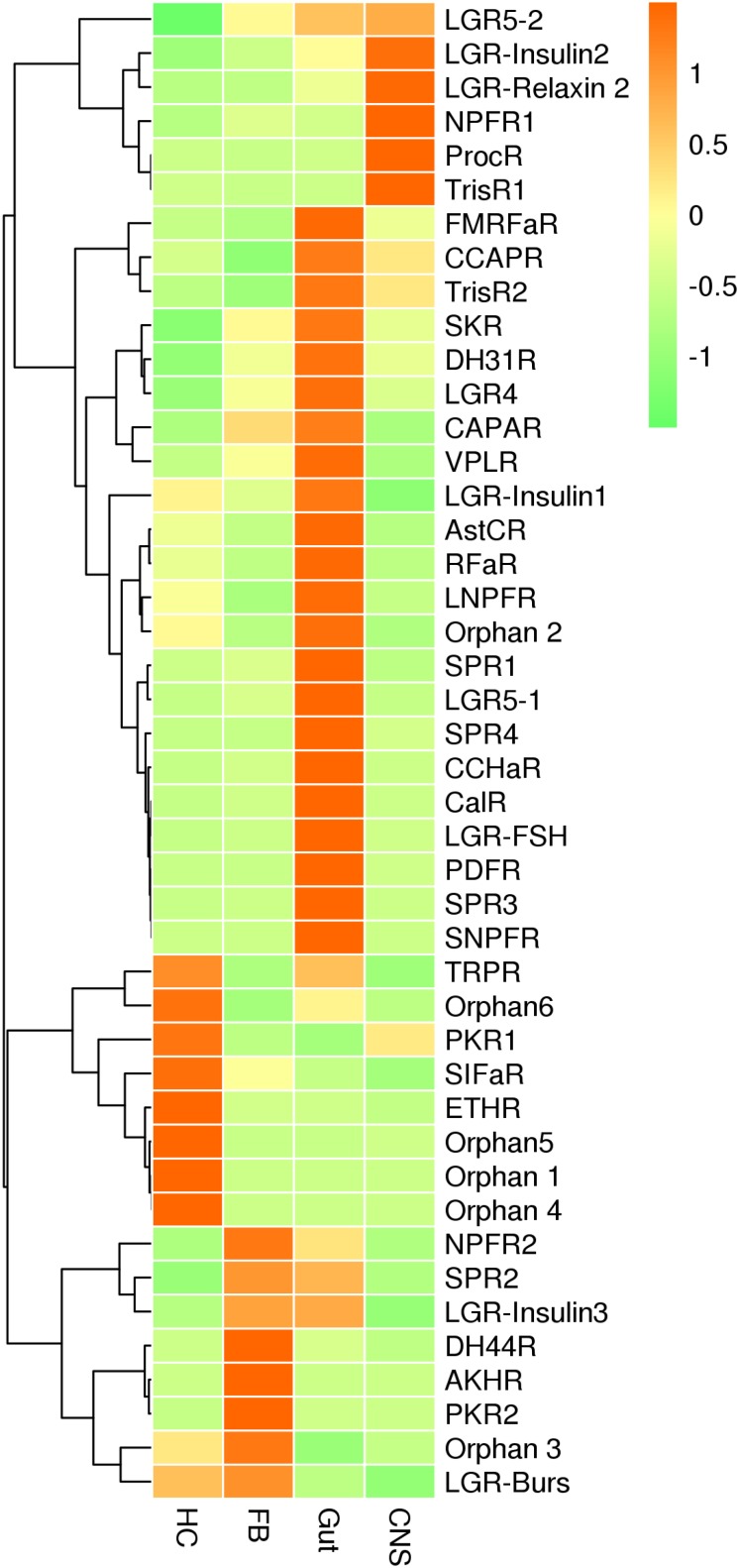
qRT-PCR results showing the relative expression levels of the neuropeptide receptors in various tissues of the red palm weevil.

For neuropeptide receptors, genes coding for receptors for ecdysis-triggering hormone and SIFamide along with four orphan GPCRs were predominately expressed in hemocytes, whereas receptors for adipokinetic hormone, pyrokinin 2, and diuretic hormone 44 were expressed at the highest levels in fat bodies. Receptors for CCHamide 1, FMRFamide, long neuropeptide F, orphan GPCR2, RFa peptide, Sex peptide 1, 3 and 4, short neuropeptide F, inotocin, calcitonin, diuretic hormone 31, pigment-dispersing factor, and insulin along with leucine-rich repeat-containing GPCR–FSH, LGR4, and LGR5-1 were mainly expressed in the gut plus Malpighian tubules. Receptors for neuropeptide F1, proctolin, and trissin-1 were predominately expressed in CNS. No differences in the remaining receptors were detected among the tissues analyzed (Figure 6 and Supplementary Figure S7).

### Impact of Pathogen Infection on the Expression of Neuropeptide and Receptor Genes

**FIGURE 7:**
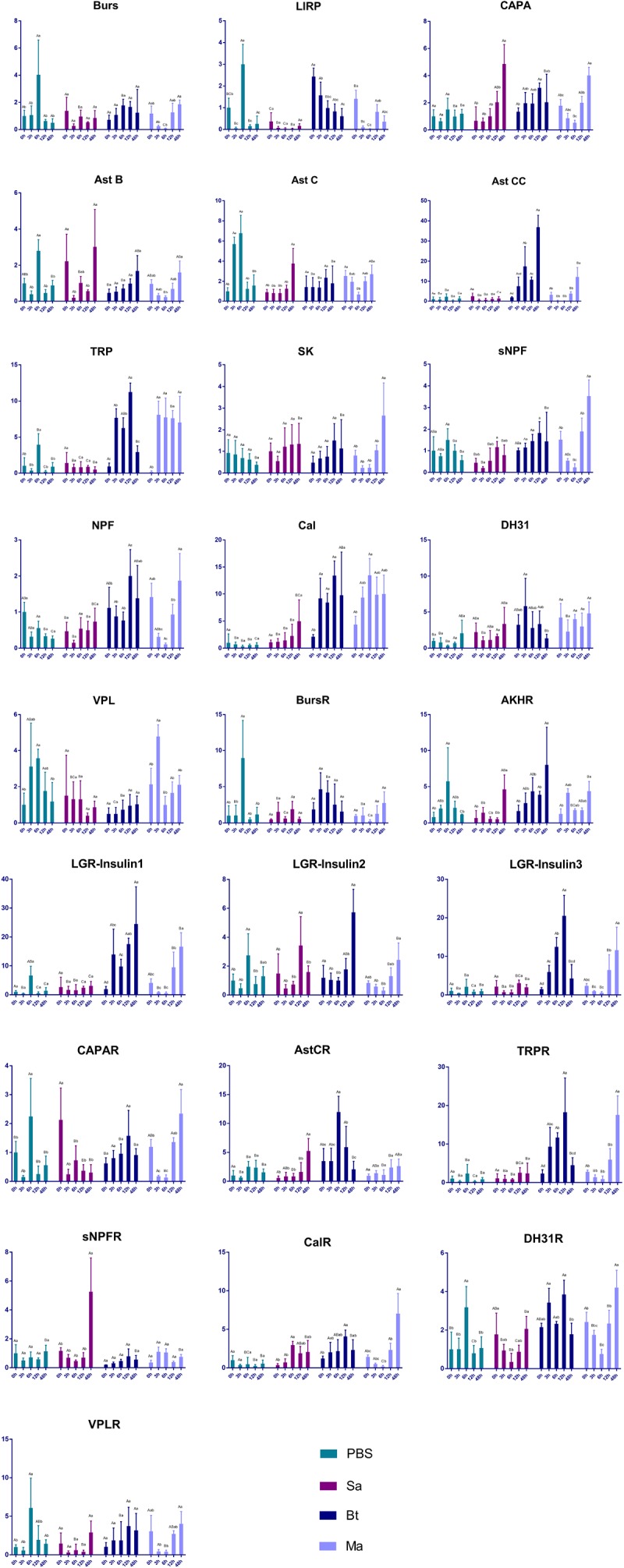
Impact of pathogen infection on the expression of neuropeptide and receptor genes. Different small letters indicate statistically significant difference among different time points for the same treatment. Different capital letters indicate statistically significant difference among different treatments of the same time point.

The expression of genes encoding neuropeptides and receptors in the red palm weevil was analyzed after the insect was infected with either *S. marcescens* (Gram−), *B. thuringiensis* (Gram+), or *M. anisopliae* (Fungus). The expression of the gene encoding the locust insulin-related peptide precursor decreased in insects infected with either *B. thuringiensis* or *M. anisopliae*. The expression of the genes encoding allatostatin CC, the GPCR for allatostatin C, tachykinin-related peptide, the receptor for tachykinin-related peptide, neuropeptide F, calcitonin, LGR-insulin 1, LGR-insulin 2, and LGR-insulin 3 increased after infection with either *B. thuringiensis* or *M. anisopliae*. The expression levels of genes encoding capability and the GPCR for short neuropeptide F increased significantly in 48 h after *S. marcescens* infection. The remaining genes were either not responsive to pathogen infection or irregularly expressed after pathogen infection (Figure 7).

## Discussion

In this study, we systematically identified neuropeptide precursors and GPCRs from the red palm weevil following a transcriptomic approach. The quality of the transcriptomes from both larvae and pupae appeared to be comparable with those from other insect species based on total numbers and average lengths of assembled unigenes, and N50 values. A total of 43neuropeptide precursors were identified from the red palm weevil, compared with 48 from *H. abietis* and 64 from *T. castaneum* (Pandit et al., 2018; Veenstra, 2019). A total of 44 neuropeptide GPCRs were identified from the red palm weevil, compared with 25 identified from *H. abietis* and 48 from *T. castaneum* (Hauser et al., 2008). The total numbers of genes coding for both neuropeptides and GPCRs were largely comparable to those from other insect species.

Despite similar numbers of neuropeptides and GPCRs identified from the red palm weevil, the repertoires of neuropeptides and GPCRs showed uniqueness in this insect species. First, two genes encoding two very different forms of myosuppressins were identified from the red palm weevil. This is striking because only one gene has been reported in most other insect species so far in the literature. Two gene encoding two myosuppresins are reported in the migratory locust, but these two neuropeptides are produced by alternative splicing and conserved at all consensus sites (Veenstra, 2014). Myosuppressins with the consensus XDVXHXFLRFamide generally play roles in regulating the gut and heart muscle contraction in insects (Nässel and Winther, 2010). Amino acid substitutions in myosuppresins can result in different developmental and tissue-specific synergetic or antagonistic effects (Dickerson et al., 2012). The two myosuppresins from the red palm weevil are very diverged, with the residues at both the 3rd and 8th positions of Val and Leu replaced with Met and Trp (Figure 1). Because of the diverged sequences, the functions for these two myosuppresin isoforms would be quite different even though the exact function of the new myosuppresin remains to be delineated.

Second, several gene expansions were observed in the red palm weevil. The most obvious expansion is the GPCRs for neuropeptide F and insulin related peptides. Neuropeptide F plays a role in regulating feeding and sleep-wake behavior of insects (Chung et al., 2017). A long and short neuropeptide F usually exist in insect species. However, four CPCRs were found in the red palm weevil. Two CPCRs correspond to the long and short NPF, respectively, but the remaining two receptors remain unknown if they are involved in similar activation pathways. Three insulin receptors were found in the red palm weevil, but only two in other insect species. The two insulin receptors in *T. castaneum* were expressed in different developmental stages, and have functionally diverged with respect to the development and reproduction (Sang et al., 2016). The newly expanded insulin receptors in the red palm weevil may also have a unique regulatory effect on its growth and development.

It is quite interesting that several neuropeptide genes were apparently not found in our study, including adipokinetic hormone, AKH/Corazonin-related peptide, agatoxin-like, crustacean cardio active peptide, hansolin, relaxin, SIFamide andRFLamide, but some of their putative corresponding receptors were identified. Similarly, GPCRs, like allatostatin B, myosuppressin and RYamide, were not found in this study, but their corresponding hormones were identified. These neuropeptides and GPCRs are ubiquitous in other Coleoptera (Veenstra, 2019). Thus most of the absence may be due to insufficient sequencing depth or incomplete annotation. However, some neuropeptides have been reported got lost repeatedly in Coleoptera, like elevenin, AKH/Corazonin-related peptide, and relaxin (Veenstra, 2019), this suggests that a few neuropeptides may have been lost if not identified. The RFLamide and receptor was not found in either of the two Curculionids, *Hypothenemus* and *Dendroctonus* (Veenstra, 2019), but the receptor was identified in the red brown weevil. So it’s still unclear whether this neuropeptide signaling is still present in the red palm weevil.

Some genes encoding neuropeptides and receptors were found to be highly expressed in tissues other than the CNS. For example, genes encoding several neuropeptides, including AstC, CCHa-2, CCAP, capa, calcitonin, FMRFamide, sNPF, myosuppressin, diuretic hormones, ion transport peptide, orcokinin and NVP-like peptide precursors, were all predominately expressed in gut plus Malpighian tubules, suggesting their important roles on feeding, digestion, diuresis, and energy homeostasis in insects. Similar observations were also found in other insect species (Audsley and Weaver, 2009; Schoofs et al., 2017). What is unique for the red palm weevil is the expression of the two genes encoding NPFs. Since NPF-genes are highly expressed in the gut of *C. suppressalis* and *L. migratoria*, it was thought that NPFs are associated with the regulation of feeding behavior (Hou et al., 2015; Xu et al., 2016). But both the long NPF and short NPF in the red palm weevil were highly expressed in the central nerve system, their corresponding receptor genes were highly expressed in gut. On the other hand, the gene encoding AstB was highly expressed in the gut of the red palm weevil larvae, but the same gene is primarily expressed in the central nerve system in *C. suppressalis* (Xu et al., 2016). These conflicting observations indicate that different insect species may regulate their signaling network differently, and there may be different recognition partners under different physiological conditions.

Neuropeptides are well-known to regulate various physiological processes and behaviors of insects, but little is known about their roles in regulating insect immunity. In this study, we found that some genes encoding neuropeptides and receptors responded to pathogen attack in the red palm weevil. The two pathogens that induced the most responses are *B. thuringiensis* and *M. anisopliae*. The induction of neuropeptide and receptor genes by pathogens indicate that these neuropeptide and receptor pairs play roles in immune response of the insect. For example, the gene encoding the tachykinin-related peptide was strongly induced in red palm weevils 3 h after *B. thuringiensis* and *M. anisopliae* infection and the effects lasted for a long time. Tachykinin-related peptide is a multifunctional neuropeptide, which may regulate insect immune system and metabolic homeostasis based on structure and functional homologs of vertebrate tachykinins (Urbanski and Rosinski, 2018). In *D. melanogaster*, intestinal microbiota and the microbial metabolite have also been found to activate innate immunity and promote the expression of the tachykinin-related peptide to promote host metabolic homeostasis (Kamareddine et al., 2018).

## Conclusion

We have systematically analyzed genes encoding neuropeptides and their corresponding receptors in the destructive pest red palm weevil via establishing a larval and a pupal transcriptome, resulting in the identification of 43 putative neuropeptide precursors and 44 neuropeptide receptors. A novel form of myosuppressin was discovered, which carries distinct amino acid residues at two conserved sites. Genes encoding neuropeptide F receptors and insulin receptors have expanded. We also analyzed the expression of the identified genes in different tissues. Some genes encoding neuropeptide precursors and receptors were highly expressed in tissues other than the CNS and may play roles other than neural signaling. Four orphan receptors may play a role in regulating immune cell activity based on their high expression in hemocytes. Moreover, some neuropeptides and receptors, like the tachykinin-related peptide and receptor, were significantly induced by pathogen infection, especially sensitive to *B. thuringiensis* and *M. anisopliae*, which may regulate insect immune system. Our research laid the foundation for future functional studies on neuropeptides and their receptors, which may lead to the development of novel pest control strategies.

## Data Availability Statement

The datasets generated for this study can be found in the NCBI GenBank, Red Palm Weevil neuropeptide receptors accession numbers: MK751489–MK751534, Red Palm Weevil neuropeptide precursors accession numbers: MK751535–MK751576.

## Ethics Statement

*Rhynchophorus ferrugineus* is exempted from above mentioned requirements.

## Author Contributions

YH and JL conceived and designed the research. HZ, JB, SH, and HL performed the experiments. HZ performed the analysis and wrote this manuscript.

## Conflict of Interest

The authors declare that the research was conducted in the absence of any commercial or financial relationships that could be construed as a potential conflict of interest.

## Footnotes

1 http://www.ncbi.nlm.nih.gov/

2 http://www.cbs.dtu.dk/services/SignalP/

3 http://weblogo.berkeley.edu/logo.cgi

4 http://www.neurostresspep.eu/diner/infosearch

5 http://tree.bio.ed.ac.uk/software/figtree

6 http://bioinfo.ut.ee/primer3-0.4.0/

## Supplementary Material

FIGURE S1 Multiple sequence alignment of short NPF **(A)** and long NPF **(B)** of a number of insect species. The sequence in the box are the predicted mature peptides and the sequence underlined in red is absent from the short NPF precursors.

FIGURE S2 Multiple sequence alignment of PDF mature peptides from a number of Coleoptera species. The sequence underlined in orange is the predicted mature peptide.

FIGURE S3 Sequence logos for the putative Orcokinin B mature peptides that are repeated in the pre-propeptide of each insects.

FIGURE S4 Multiple sequence alignment of the putative CCHa-1 mature peptides.

FIGURE S5 Multiple sequence alignment of the putative CCHa-2 mature peptides.

FIGURE S6 qRT-PCR histogram results showing the relative expression levels of the neuropeptide precursors in various tissues of the red palm weevil. The expression levels were estimated using the 2^–Δ^ ^Δ^ ^CT^ method. Bars represent standard error of four independent biological replicates with three technical duplicates for each replicate. Different small letters indicate statistically significant difference between tissues (*p* < 0.05, ANOVA, HSD).

FIGURE S7 qRT-PCR histogram results showing the relative expression levels of the neuropeptide receptors in various tissues of the red palm weevil.

TABLE S1 List of neuropeptide genes of red palm weevil.

TABLE S2 List of neuropeptide receptor genes of red palm weevil.

TABLE S3 Putative Neuropeptides/Neuropeptide-Like Peptides from precursors of red palm weevil.

TABLE S4 Neuropeptide genes in the red palm weevil and other Coleopteran beetles. √, identified; ×, not identified.

TABLE S5 Primers of candidate neuropeptide precursors and receptors in used for RT-qPCR.
