# Supplementary figures and images for: Neuropeptides and G-Protein Coupled Receptors (GPCRs) in the Red Palm Weevil Rhynchophorus ferrugineus Olivier (Coleoptera: Dryophthoridae)
Source: Front Physiol. 2020 Feb 28;11:159. doi: 10.3389/fphys.2020.00159 (PMC7058690; doi:10.3389/fphys.2020.00159)

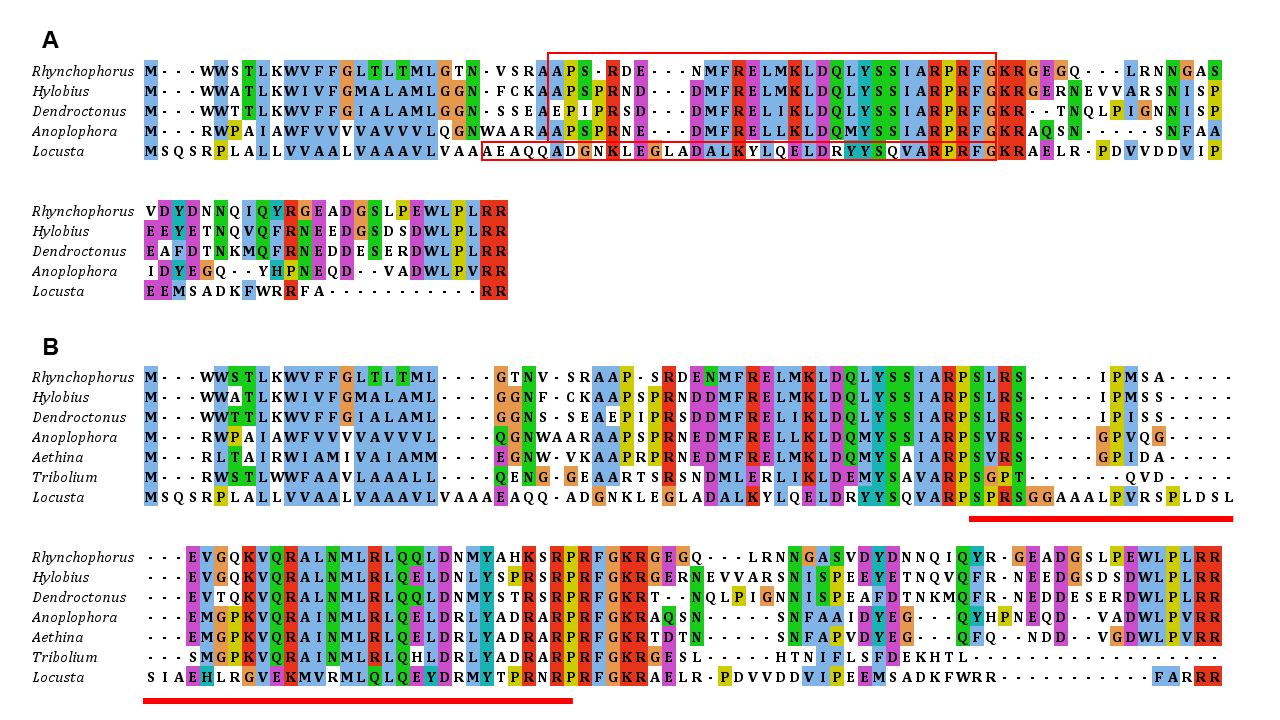

Supplement: FIGURE S1 — Multiple sequence alignment of short NPF (A) and long NPF (B) of a number of insect species. The sequence in the box are the predicted mature peptides and the sequence underlined in red is absent from the short NPF precursors. [file Image_1.PNG]

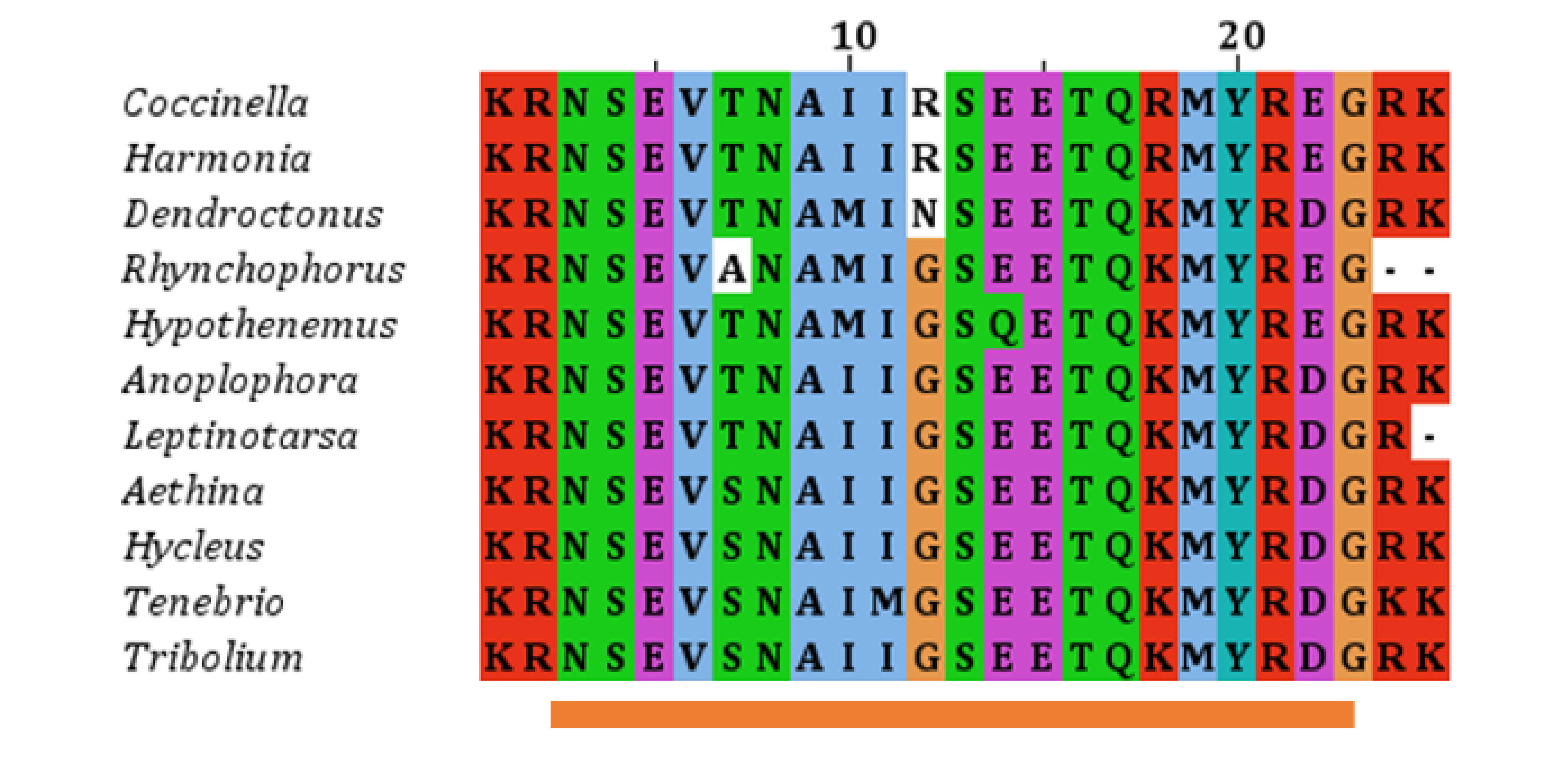

Supplement: FIGURE S2 — Multiple sequence alignment of PDF mature peptides from a number of Coleoptera species. The sequence underlined in orange is the predicted mature peptide. [file Image_2.TIF]

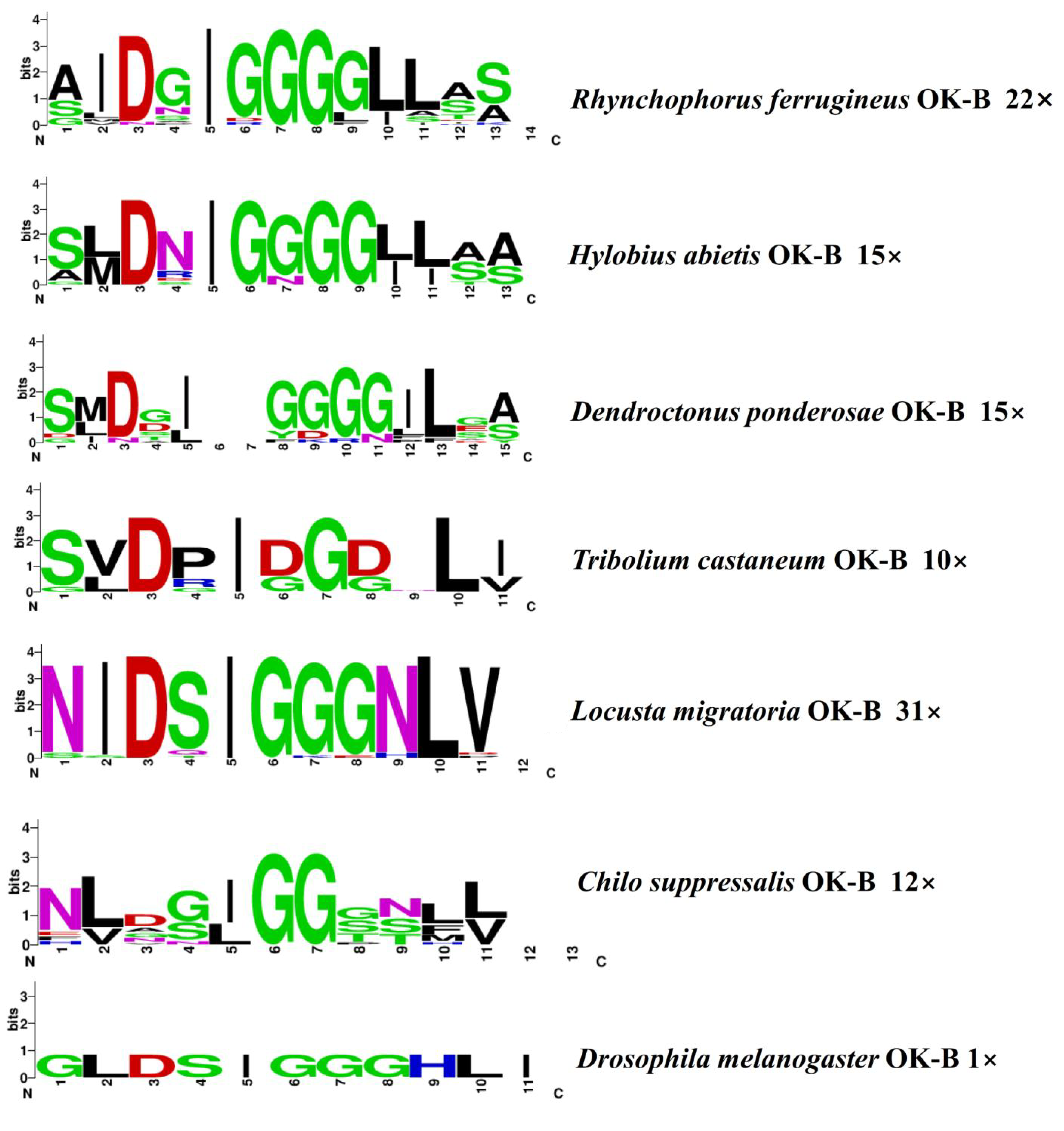

Supplement: FIGURE S3 — Sequence logos for the putative Orcokinin B mature peptides that are repeated in the pre-propeptide of each insects. [file Image_3.TIF]

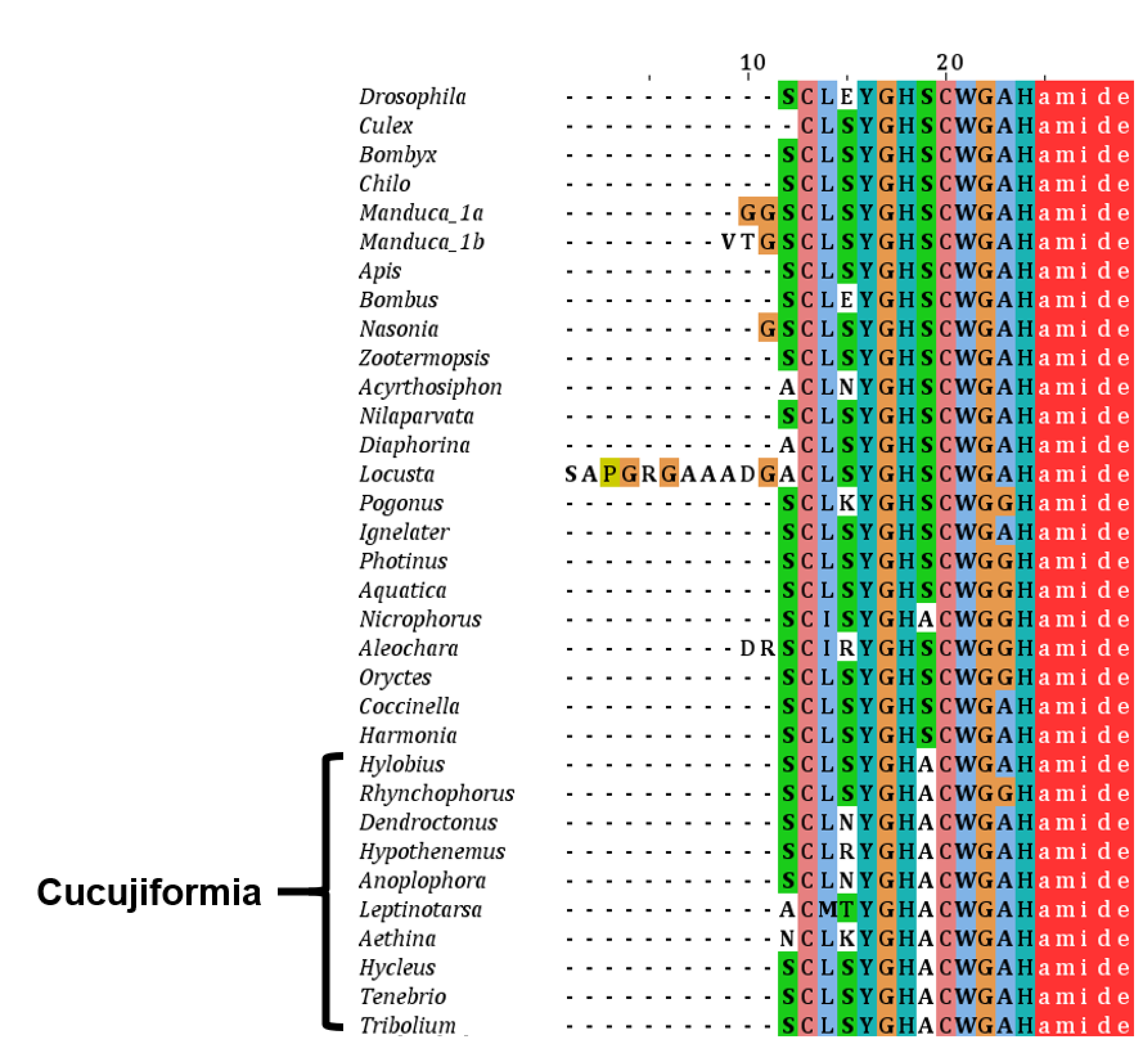

Supplement: FIGURE S4 — Multiple sequence alignment of the putative CCHa-1 mature peptides. [file Image_4.TIF]

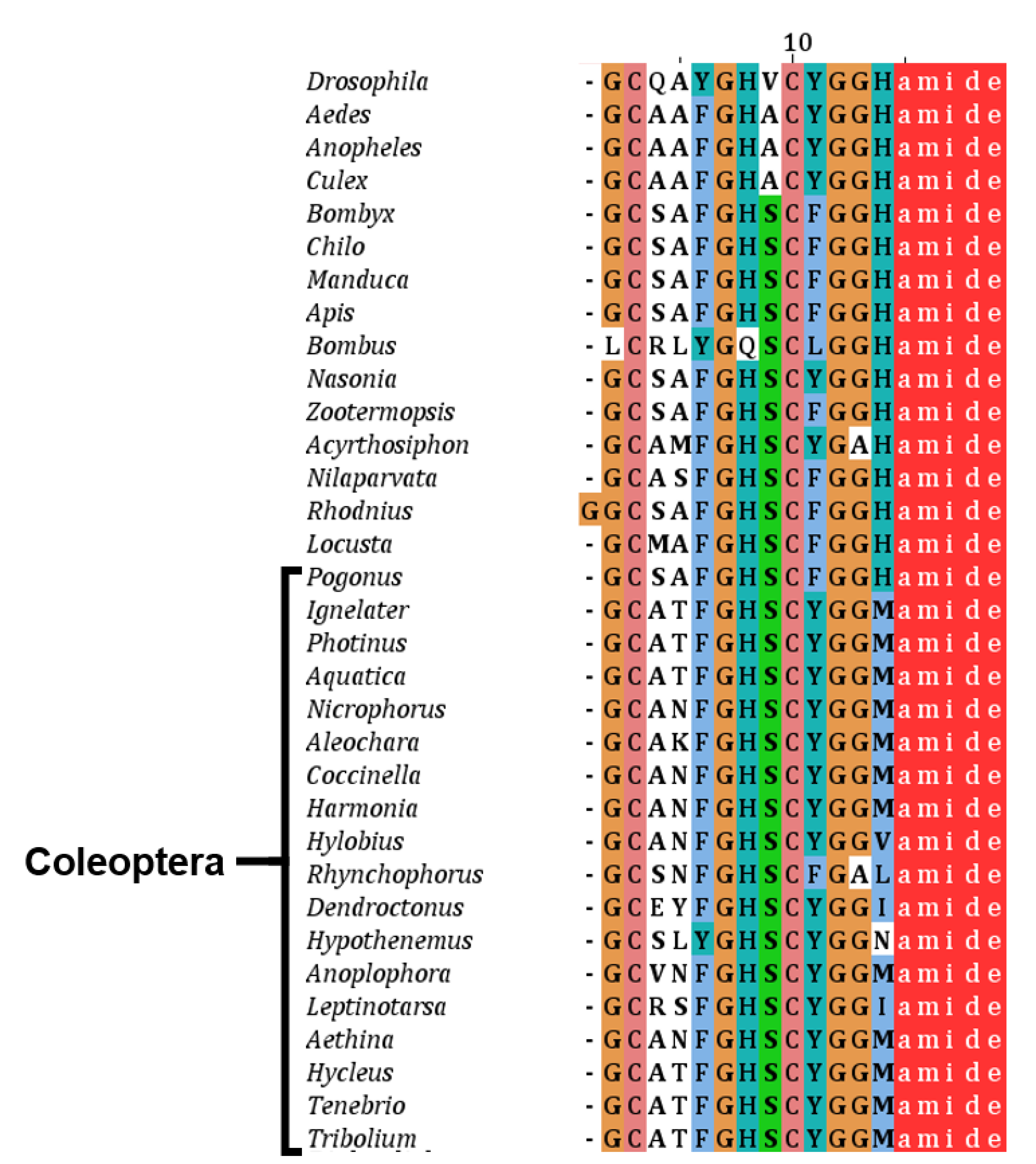

Supplement: FIGURE S5 — Multiple sequence alignment of the putative CCHa-2 mature peptides. [file Image_5.TIF]

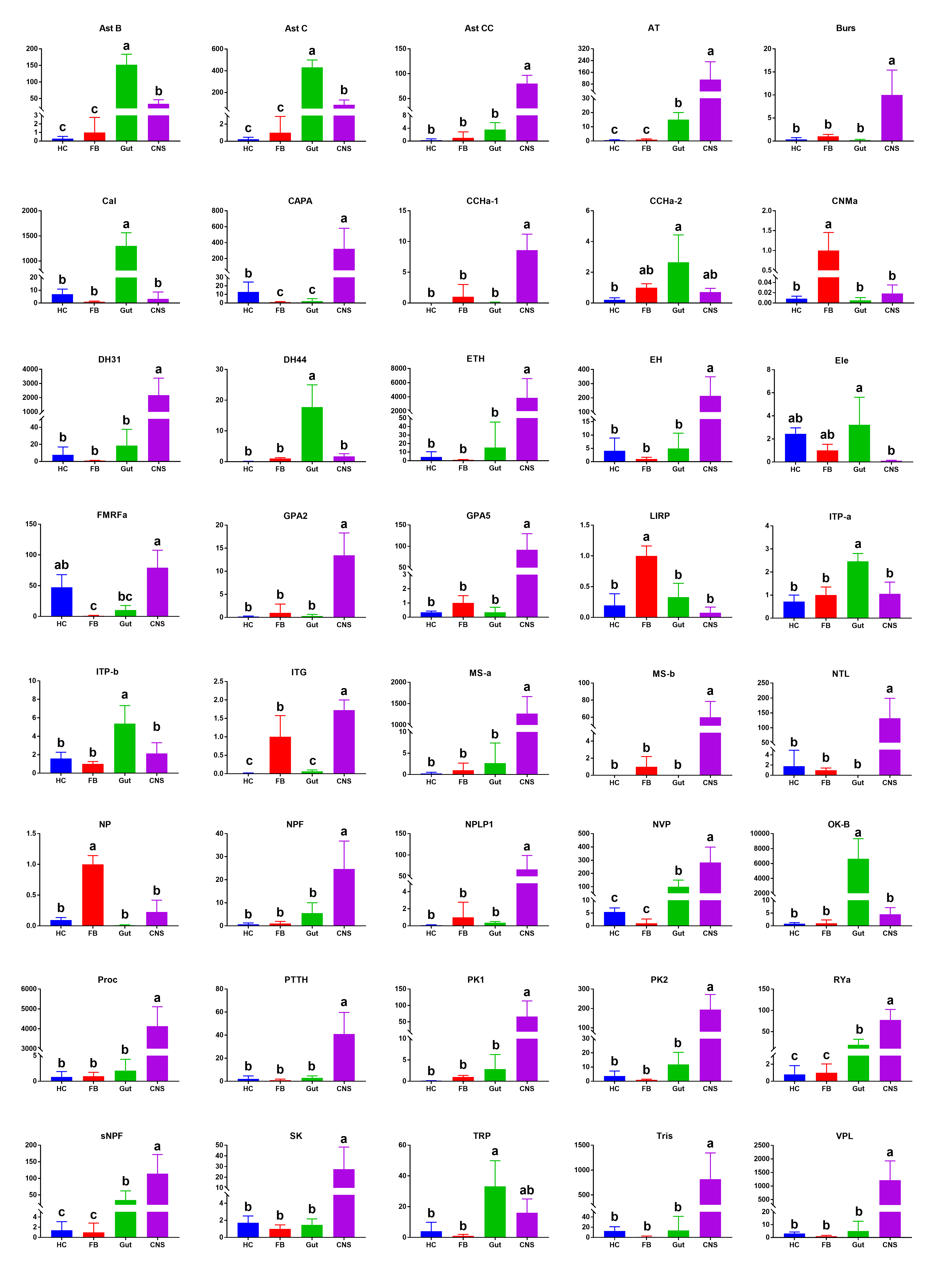

Supplement: FIGURE S6 — qRT-PCR histogram results showing the relative expression levels of the neuropeptide precursors in various tissues of the red palm weevil. The expression levels were estimated using the 2–Δ Δ CT method. Bars represent standard error of four independent biological replicates with three technical duplicates for each replicate. Different small letters indicate statistically significant difference between tissues (p < 0.05, ANOVA, HSD). [file Image_6.TIF]

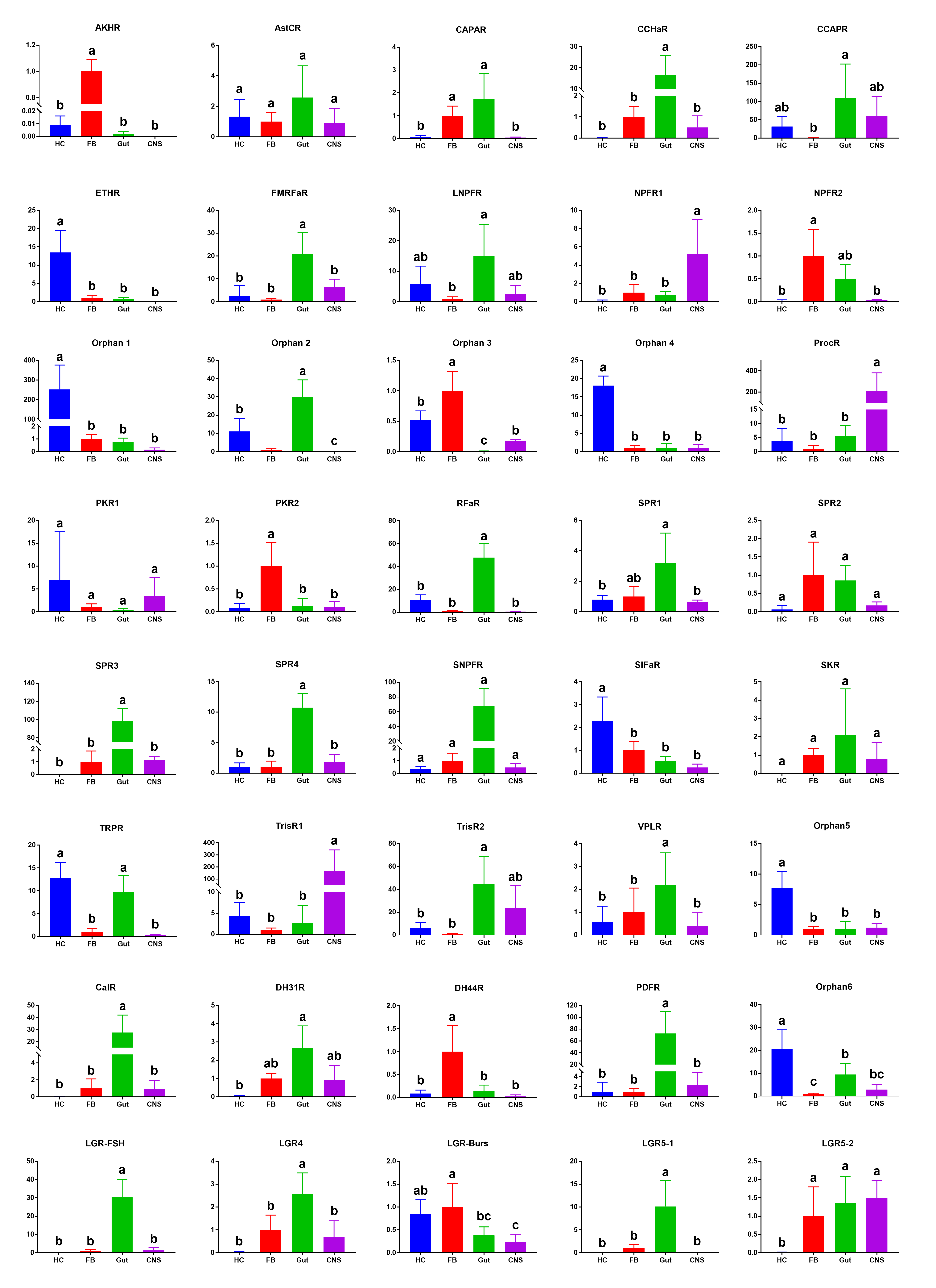

Supplement: FIGURE S7 — qRT-PCR histogram results showing the relative expression levels of the neuropeptide receptors in various tissues of the red palm weevil. [file Image_7.TIF]

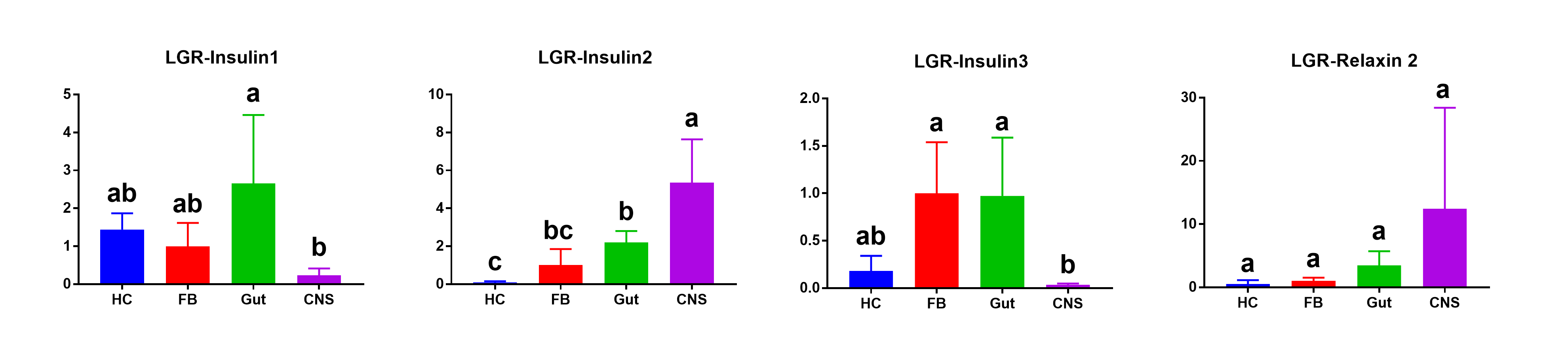

Supplement: Supplementary file 8 [file Image_8.TIF]
